# Population Pharmacokinetics of Vancomycin in Pregnant Women

**DOI:** 10.3389/fphar.2022.873439

**Published:** 2022-06-06

**Authors:** Rahul K. Goyal, Brady S. Moffett, Jogarao V. S. Gobburu, Mayar Al Mohajer

**Affiliations:** ^1^ University of Maryland, Baltimore, MD, United States; ^2^ Texas Children’s Hospital, Houston, TX, United States; ^3^ Baylor College of Medicine, Houston, TX, United States

**Keywords:** vancomycin, pregnancy, therapeutic drug monitoring, population pharmacokinetic (PK) model, obsterics, antibiotics

## Abstract

**Objective:** Vancomycin is a glycopeptide antibacterial indicated for serious gram-positive infections. Pharmacokinetics (PK) of vancomycin have not been described in pregnant women. This study aims to characterize the PK disposition of vancomycin in pregnant women based on data acquired from a database of routine hospital care for therapeutic drug monitoring to better inform dosing decisions.

**Methods:** In this study, plasma drug concentration data from 34 pregnant hospitalized women who were administered intravenous vancomycin was analyzed. A population pharmacokinetic (PPK) model was developed using non-linear mixed effects modeling. Model selection was based on statistical criterion, graphical analysis, and physiologic relevance. Using the final model AUC_0-24_ (PK efficacy index of vancomycin) was compared with non-pregnant population.

**Results:** Vancomycin PK in pregnant women were best described by a two-compartment model with first-order elimination and the following parameters: clearance (inter individual variability) of 7.64 L/hr (32%), central volume of 67.35 L, inter-compartmental clearance of 9.06 L/h, and peripheral volume of 37.5 L in a typical patient with 175 ml/min creatinine clearance (CRCL) and 45 kg fat-free mass (FFM). The calculated geometric mean of AUC_0-24_ for the pregnant population was 223 ug.h/ ml and 226 ug.h/ ml for the non-pregnant population.

**Conclusion:** Our analysis suggests that vancomycin PK in pregnant women is consistent with non-pregnant adults and the dosing regimens used for non-pregnant patients may also be applicable to pregnant patients.

## Introduction

Vancomycin is a glycopeptide antibacterial indicated for the treatment of serious Gram-positive infections; for e.g., infections caused by methicillin-resistant *Staphylococcus aureus* (Rybak et al., 2020). Although vancomycin is widely used in hospitals, there is no consensus among clinicians with regard to dosing regimens of vancomycin and therapeutic drug monitoring (TDM) is suggested due to two reasons (Ingram et al., 2008). First, under-dosing of vancomycin causes drug resistance and loss of effectiveness, whereas over-dosing causes serious adverse effects, such as nephrotoxicity and ototoxicity (Bruniera et al., 2015; Filippone et al., 2017). Second, vancomycin is associated with large inter-individual variability (IIV) in the pharmacokinetic (PK) parameters (Aljutayli et al., 2020). Nevertheless, a myriad of population pharmacokinetic (PPK) models have been developed to describe vancomycin disposition and inform suitable dosing regimens to achieve necessary PK endpoints i.e., attainment of goal serum concentrations and area under the curve (AUC) to minimum inhibitory concentration (MIC) ratio of >400 (Rybak et al., 2020). PPK models are commonly used to identify dosing regimens that are most optimal for achieving a therapeutic target before starting dosing. More recently, for TDM drugs such as vancomycin that have narrow therapeutic index and are highly variable, especially in heterogenous populations such as pediatrics, PPK models are also being used to inform precision dosing (Frymoyer et al., 2020; Heine et al., 2020). With the advent of clinical decision support tools, such as Lyv software, model informed precision dosing approaches can tailor treatment trajectories spontaneously (Jarugula et al., 2021). While most of the PPK models for vancomycin were investigated in different sub-populations including geriatrics, pediatrics and obese patients, vancomycin pharmacokinetics have not been described in pregnant women.

Vancomycin is not specifically labeled for use in pregnant population and studies published in literature indicate that vancomycin is not teratogenic at therapeutic concentrations (Reyes et al., 1989). Hence, prescribers typically use the same dosing regimens that are approved for non-pregnant patients. However, pregnancy is associated with physiological changes and altered drug PK (Widen and Gallagher, 2014; Feghali et al., 2015). Vancomycin is 55% bound to proteins, widely distributed into body tissues, and primarily eliminated by kidney (Moellering, 1984; Matzke et al., 1986) all of which might be altered in pregnant women. Knowledge of the PK behavior of vancomycin in pregnancy is necessary to ensure that dosing is appropriate for this special population and endpoints of interest are met. The main objective of this study was to characterize the PK of vancomycin in pregnant population to better inform the dosing decisions in clinical practice. To that end, a PPK model was developed and covariates significant for alterations in vancomycin PK were identified.

## Materials and Methods

### Patients and Data Collection

This was a retrospective PPK study for which Institutional Review Board approval was obtained (IRB# H-46182). The Texas Children’s Hospital electronic medical record was queried from 1 January 2011—31 May 2019, to obtain data collected during routine patient care of TDM. Patients were included in the dataset if they were admitted and discharged as an inpatient during the study period and were administered intravenous vancomycin; and have had one or more vancomycin serum concentrations sampled and measurable. Exclusion criteria consisted of patients who were receiving extracorporeal renal replacement therapy (continuous renal replacement, peritoneal dialysis, hemodialysis) concomitantly with vancomycin, concomitant administration of vancomycin by a route other than intravenous, or patients who had vancomycin administered prior to admission.

Along with vancomycin dose and serum concentrations, covariates included as a part of the dataset were demographic variables–patient age, total body weight (TBW), height, gestational age, patient serum creatinine values, serum creatinine sample date and time. Creatinine clearance (CRCL), fat-free mass (FFM), and body mass index were derived covariates. CRCL was calculated by the modified Schwarz equation for patients <19 years of age and the Cockroft-Gault equation for patients ≥19 years of age. FFM was calculated using the formula from Al-Sallami et al. (Al-Sallami et al., 2015) for patients <18 years of age and the formula from Janmahasatian et al. (Janmahasatian et al., 2005) for patients ≥18 years old.

### Blood Sampling

Vancomycin serum concentrations were collected in either a 1 × 0.6 ml Amber Microtainer with Gel or 1 × 1 ml Red/Black Serum Separator Vacutainer. The vancomycin assay was performed by using the VITROS Chemistry Products VANC Reagent in conjunction with the VITROS Chemistry Products Calibrator Kit 11 on the VITROS 5600 Integrated System (Ortho Clinical Diagnostics, Raritan, NJ). The assay was based on competition between vancomycin in the sample and vancomycin labeled with Glucose-6-phosphate dehydrogenase (G6P-DH) for antibody binding sites. Activity of G6P-DH decreases upon binding to the antibody; therefore, vancomycin concentration in the sample can be measured in terms of G6P-DH activity. The analytic measurement range was 5–50 mg/L. The coefficient of variation was <6%.

### Data Analysis

All analyses were conducted using Pumas 2.0 (Pumas-AI, Baltimore) (Rackauckas et al., 2020). Non-linear mixed effects modeling approach using second order Laplace approximation with interaction was applied to characterize the PK disposition of vancomycin in pregnant women. A hierarchical model building approach was opted. A two-compartment model that was built on non-pregnant adults was used as a base model. Covariates were added sequentially if they supported explanation of the variability of PK parameters.

### Pharmacokinetic Modeling

The modeling approach is motivated by a previous research project by taking advantage of models from literature for the choice of a base model, and, in addition using biological relevance for covariate modeling (Pastoor, 2019). Different two-compartment models that were identified from literature search by and large contained either one or both of CRCL and TBW as covariates on clearance and volume parameters (Thomson et al., 2009; Aljutayli et al., 2020). The base model has been modified to contain CRCL and FFM as covariates on clearance of central compartment (CL), and FFM as a covariate on volume of central compartment (V_c_), volume of peripheral compartment (V_p_), and inter-compartmental clearance (Q) (Supplementary Table S1). Using the modified base model, concentrations for the pregnant population were predicted using empirical Bayes’ estimation. The model was qualified to be a suitable choice of base model by visual inspection of goodness-of-fit (GOF) plots and individual PK profiles. After qualifying the base model, model fitting was performed for the pregnant.

As data was collected from a TDM database, most of the concentration samples in the dataset were trough concentrations. Therefore, reasonably precise estimation of all parameters was not feasible. Selection of variance components in the model was based on physiological relevance, shrinkage, and absolute value. A variance component was dropped if it was either too small or too large and/or the associate shrinkage was greater than 30%. Moreover, V_p_, Q, and the exponent on CRCL were fixed to the values based on published literature and were adjusted to account for the difference in the choice of covariate model containing FFM instead of TBW.

A truncated error model (commonly known as the M2 method) was used by specifying the lower limit of quantification (LLQ) as 5 mg/L and the upper limit as infinity (Beal, 2001). Based on random effect (eta) versus covariate plots, all covariates that can potentially explain the IIV for all the parameters were explored. Each covariate was tested to be included based on eta versus covariate plots to develop the final model. The variance components were tested to be reasonably distributed around zero.

### Model Selection and Evaluation

The final model was selected based on physiological relevance, log-likelihood value (OFV), Bayesian information criterion (BIC), and graphical analysis. GOF plots, such as observed concentration (DV) versus predicted concentration (IPRED), conditional weighted residuals (CWRES) versus population predicted concentration (PRED), and CWRES versus time after dose (TAD) were inspected for model diagnostics. Lastly, individual observed, and predicted PK profiles were also a part of visual evaluation. Bootstrap simulations with 1,000 samples with replacement was carried out on the final model for the re-estimation of parameters and building 95% confidence intervals.

### Comparison With Non-Pregnant PPK Model

The accepted pharmacokinetic/pharmacodynamic index is for AUC/MIC ratio to be > 400 (Rybak et al., 2020) and hence geometric mean of AUC_0-24_ was chosen as a PK endpoint to compare pregnant and non-pregnant population. Using the dosing regimen and patient characteristics of the 34 subjects from the current dataset, concentration-time data were generated using the non-pregnant model and the final model developed in this study. AUC_0-24_ was calculated using non-compartmental analysis to compare the exposures obtained from these two models.

## Results

### Patients and Data Collection Summary (Demographics)

Patient demographics and baseline characteristics are summarized in Table 1. A total of 91 samples that were collected across 34 subjects. Nine samples were below the limit of quantification (5 mg/L) and were excluded from the final dataset used for modeling. Majority of the samples were trough samples with at least half of them collected within 2 h prior to dose administration. 22 subjects had normal kidney function at baseline with serum creatinine between 0.4 and 0.8 mg/dl (35.3–70.7 μmol/L). Three subjects had serum creatinine below 0.4 mg/dl and 9 subjects had serum creatinine above 0.8 mg/dl. There were two subjects in the first trimester of pregnancy, 15 in the second trimester, and 17 in the third trimester. The median (IQR) total daily dose was 3,000 mg (2000–4,000 mg).

**TABLE 1:** Patient demographics and baseline characteristics.

Variable	Value^a^
Number of patients	34
Age (years)	28 (17–38)
Height (cm)	163 (147–173)
Total body weight (kg)	74 (43–157)
Gestational age (weeks)	27 (7–40)
Serum creatinine (mg/dl)	0.56 (0.27–1.97)
Creatinine clearance^b^ (ml/min)	176 (43–389)
Fat-free mass^c^ (kg)	45 (30–60)
Body mass index (kg/m^2^)	28 (19–70)

a Results are presented as median (range).

b Creatinine clearance calculated using Cockroft-gault equation for patients >19 years and Modified Schwartz equation for patients <19 years of age.

c Lean body mass is calculated by using Janmahasatian et al. for patients >18 years of age and Al-Sallami et al. for patients <18 years of age.

### Population Modeling

**FIGURE 1:**
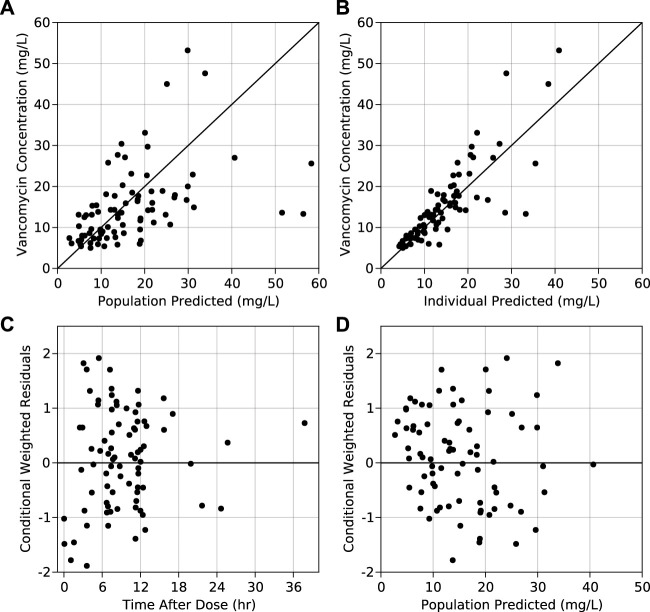
Observed vs. **(A)** population predicted concentrations and **(B)** individual predicted concentrations obtained from the final model. Conditional weighted residuals obtained from the final model vs. **(C)** time after dose and **(D)** population predicted concentration from the final model.

A two-compartment model with first-order elimination best described vancomycin PK in pregnant women. Among all the IIVs, central compartment variance components were prioritized for estimation over visceral parameters as they are of higher clinical importance. The shrinkage associated with V_c_, V_p_, and Q was >90%. Upon stepwise elimination of the variance components, in the end, IIV was estimable only for CL. Also, histogram of IIV of CL showed that it is reasonably distributed around zero (Supplementary Figure S1). Among the population parameters, V_p_, Q, and the exponent on CRCL were fixed to the values from the non-pregnant model. Individual post-hoc estimates of the IIV on CL from the base model versus covariates did not show significant correlation (Supplementary Figure S2), and hence the base model was chosen as the final PPK model. GOF plots of the final model are shown in Figure 1 (and in log scale in Supplementary Figure S3). Final model code is provided in the supplementary data to enable reproducibility (Supplementary Code S1).

**FIGURE 2:**
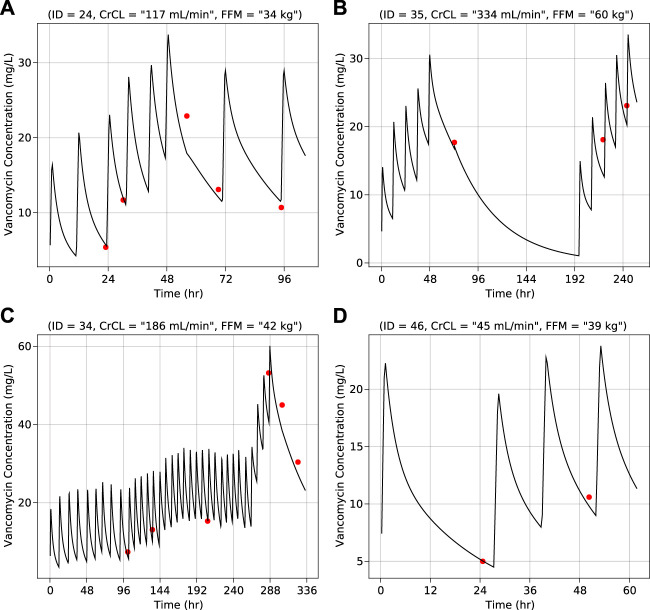
Vancomycin concentration vs. time for individual representative subjects–**(A)** Normal creatinine clearance and low fat-free mass **(B)** High creatinine clearance and high fat-free mass **(C)** Normal creatinine clearance and normal fat-free mass **(D)** Low creatinine clearance and normal fat-free mass. Lines (black) represent predicted concentrations and dots (red) represent observed concentrations. Values of creatinine clearance and fat-free mass are at baseline.

Results of the bootstrap simulation performed 1,000 times along with the final PK parameter estimates are displayed in Table 2. The median estimate from bootstrap were same as the estimates of the final PK model and lied in the 95% confidence interval demonstrating the stability of the final PK model. Representative individual subject plots are displayed in Figure 2 for patients with low, medium, and high baseline FFM and CRCL. The close alignment between the predicted and observed concentrations indicated acceptable model accuracy. The geometric mean (IQR) of AUC_0-24_ calculated using the pregnant and non-pregnant model estimates were 223 μg h/ml (170 μg h/ml—273 μg h/ml) and 226 μg h/ml (178 μg h/ml—290 μg h/ml) respectively.

**TABLE 2:** Final PPK model parameter estimates.

Final PK Model				Bootstrap	
Parameter	Formula	Estimates	IIV in CV% [shrinkage]	Estimates	95% Confidence Interval
CL (L/h)	CL. (CRCL/175)^θCRCL^. (FFM/45)^0.75^	7.64	31.9 [0.21]	7.64	6.38–9.73
*θ*_CRCL_		1.0 (Fixed)	-	1.0 (Fixed)	NE
V_c_ (L)	V_c_. (FFM/45)	67.35	NE	67.35	41.96–112.95
Q (L/h)	Q. (FFM/45)^0.75^	9.06 (Fixed)	NE	9.06 (Fixed)	NE
V_p_ (L)	V_p_. (FFM/45)	37.5 (Fixed)	NE	37.5 (Fixed)	NE
Proportional Error (%) [shrinkage]		32.1 [0.21]		32.1	18.1–45.8

## Discussion

Due to risk to the mother and fetus, pregnant women are usually excluded from well-controlled clinical trials, thereby knowledge gaps of drug dispositions in pregnant population are higher as compared to other populations (Blehar et al., 2013; Shields and Lyerly, 2013). Hence, safe and effective use of most antibiotics in pregnant population is not known (Mitchell et al., 2011; Bookstaver et al., 2015). Vancomycin is known to cross placenta and its presence has been detected in the amniotic fluid (Bourget et al., 1991). However, a study in pregnant women who were administered vancomycin at routine doses reported that vancomycin does not cause teratogenicity (Reyes et al., 1989). An understanding of vancomycin PK during pregnancy can support effective usage of vancomycin in the clinic and guarantee that the necessary endpoints are being met. To our knowledge, this is the first reported study to-date to explain the PK disposition of vancomycin in pregnant women using a PPK approach.

Vancomycin pharmacokinetics have been widely reported to follow a two-compartment model with first-order elimination (Aljutayli et al., 2020). Additionally, it is a hydrophilic drug (log P of −3.1) and is mostly renally cleared (∼80%) (Matzke et al., 1986). Thus, FFM and CRCL were chosen as covariates in the model. The same model was then used to estimate the PK parameters for pregnant population. It must be noted that most studies have chosen TBW as a covariate in their analyses (Aljutayli et al., 2020). TBW was tested as a covariate in our analysis which resulted in a 12-point increase in the objective function value (OFV) as compared to FFM, and the IIV on CL was approximately 8% lower for the model with FFM. Therefore, both by statistical criteria and biological relevance, FFM was deemed to be a significant covariate in the final model.

Renal function, as reflected by CRCL, directly influences the elimination of vancomycin. The CRCL is known to increase beyond 120–140 ml/min during pregnancy (Dallmann et al., 2017; Lopes van Balen et al., 2019) which was also the case in our dataset (Table 1). A known limitation of the Cockroft-gault equation is that it could lead to CRCL estimates that are physiologically implausible in normal renal function subjects. Pharmacokineticists have been capping these higher CRCL estimates at about 120–140 ml/min. As the physiologic homeostasis levels of glomerular filtration in pregnant women increase, the CRCL was not capped for the final analyses. To be thorough, a sensitivity analyses was also conducted by capping the CRCL at 120 ml/min and in another scenario at 150 ml/min (Llopis-Salvia and Jiménez-Torres, 2006; Wilhelm and Kale-Pradhan, 2011; Winter et al., 2012; Li et al., 2021). For the final model, the individual clearance values scaled proportionally to the CRCL, without any tendency to plateau. The models with a capped CRCL resulted in increased variability at the capped estimate, to a comparable range as the final model. In addition, the capped models led to an over-estimation of clearance throughout the range of CRCL to compensate for the large dispersion of clearances at the higher end. This bias could yield higher vancomycin doses than necessary if CRCL is capped. These observations were the primary basis for supporting the choice of the model without capping as the final model. Further, the OFV value for the final model (472) was significantly lower than those when CRCL was capped at 120 ml/min (483) or 150 ml/min (489).

Although 10% of the samples in the dataset below LLQ were excluded, a truncated error model (M2 method) was used by specifying LLQ as 5 mg/L to reduce bias in the estimation of PPK parameters (Beal, 2001). Additionally, there is a purported role of albumin levels in the PK of vancomycin, particularly in pregnant women (Dallmann et al., 2017). Low albumin levels can result in higher concentrations of free unbound drug that in-turn might lead to a reduced volume of distribution. However, albumin in not routinely monitored in a hospital setting. For this reason, albumin could not be tested as a potential covariate in our analysis. It might be interesting to evaluate the role of albumin in future investigations to test an unbound drug target approach (Leroux et al., 2019).

The estimate of CL for a typical subject of 45 kg FFM and 175 ml/min CRCL in the pregnant population (7.64 L/h) is similar to typical CL of an equivalent subject in the non-pregnant population (9.9 L/h) calculated using the formula for CL from the non-pregnant model (Supplementary Table S1), whereas the typical estimate of V_c_ for a typical subject of 45 kg FFM in the pregnant population (67.3 L/h) is moderately higher than typical V_c_ of an equivalent subject in non-pregnant population (37.5 L/h) calculated using the formula for V_c_ from the non-pregnant model (Supplementary Table S1). The estimate of approximately 80% higher volume in the pregnant could be explained by the altered physiological changes in pregnant women. For example, there is an increase in the amount of total body water, blood volume, and capillary hydrostatic pressure during pregnancy (Costantine, 2014). Also it is to be noted that vancomycin crosses the placenta and is detected in amniotic fluid (Bourget et al., 1991) which further explains possibility for an increased V_c_. Lastly, the study dataset mostly included trough samples which also limits the ability to precisely estimate V_c_ (95% CI: 41.96–112.95 L). On the other hand, the precision of the estimate for CL parameter was satisfactory (CI: 6.38–9.73 L/h).

It is interesting to observe that the calculated AUC_0-24_ is less than 400 μg h/ml which is generally accepted PK index for efficacy assuming an MIC of 1 mg/L, however the therapeutic target at the time of dosing was not based on achieving a particular AUC but was rather based on achieving a target trough concentration between 5 and 20 mg/L. The calculated median (IQR) of individual predicted trough concentration across all the 34 pregnant subjects and dosing occasions was 10.1 mg/L (7.0 mg/L—14.5 mg/L). Nevertheless, PK can be compared because the model and its parameters are independent of the therapeutic target used for dosing implying that should the dosing regimen be designed to achieve a particular AUC in pregnant women the expected PK might be like non-pregnant population. In conclusion, our analysis showed that the calculated geometric mean of AUC_0-24_ using the pregnant model (223 μg h/ml) is commensurate with the geometric mean of AUC_0-24_ calculated using the non-pregnant model (226 μg h/ml) suggesting that dosing regimens used for non-pregnant patients may also be applicable to pregnant patients.

## Data Availability

The datasets presented in this article are not readily available because This dataset is proprietary information belonging to Texas Children's Hospital. Requests to access the datasets should be directed to jobburu@rx.umaryland.edu.
