# Supplementary material for: Population Pharmacokinetics of Vancomycin in Pregnant Women
Source: Front Pharmacol. 2022 Jun 6;13:873439. doi: 10.3389/fphar.2022.873439 (PMC9207242; doi:10.3389/fphar.2022.873439)
Supplement: Supplementary file 1 [file DataSheet1.docx]

**Supplementary Table 1.** Base model and parameters

| Parameter | Formula | Population Typical Value | IIV in CV% |
| --- | --- | --- | --- |
| CL (L/h) | CL . (CRCL/80)^θCRCL^ . (FFM/6)^0.75^ | 1.0 | 51.4 |
| θ_CRCL_ |  | 1.0 | - |
| V_c_ (L) | V_c_ . (FFM/6) | 5.0 | 41 |
| Q (L/h) | Q . (FFM/6)^0.75^ | 2.0 | 40 |
| V_p_ (L) | V_p_ . (FFM/6) | 5.0 | 20.8 |
| Proportional Error (%) |  | 7.0 |  |

**Supplementary Figure 1.** Histogram of the empirical Bayes estimate of clearance (ηCL) obtained from the final model

**Supplementary Figure 2.** Empirical Bayes estimate of clearance (ηCL) obtained from the final model vs. (a) Age (b) Gestational age (c) Height (d) Creatinine clearance (e) Total body weight (f) Fat-free mass.

**Supplementary Figure 3.** Observed vs. (a) population predicted concentrations and (b) individual predicted concentrations obtained from the final model in log scale

**Supplementary Code 1.** Pumas code of the final model for fitting

using Pumas

data4nlme = read_pumas(pkdata, observations = [:dv],

covariates = [:Age_yrs, :Wt_kg, :Ht_cms,

:GA, :CrCL, :FFM])

nlme_model = @model begin

@param begin

tvcl ∈ RealDomain(lower = 0)

tvvc ∈ RealDomain(lower = 0)

tvvp ∈ RealDomain(lower = 0)

tvq ∈ RealDomain(lower = 0)

exp_crcl ∈ RealDomain(lower = 0)

ΩCL ∈ RealDomain(lower = 0)

σ_prop ∈ RealDomain(lower = 0.0001)

end

@random ηCL ~ Normal(0, ΩCL)

@covariates CrCL FFM

@pre begin

CL = tvcl * (FFM/45)^0.75 * (CrCL/175)^exp_crcl * exp(ηCL)

Vc = tvvc * (FFM/45)

Vp = tvvp * (FFM/45)

Q = tvq * (FFM/45)^0.75

end

@dynamics Central1Periph1

@derived begin

cp := @. Central/Vc

dv ~ @. truncated(Normal(cp, abs(cp) * σ_prop), 4.99999, Inf)

end

end

init_params = (tvcl = 7.0, tvvc = 37.0, tvvp = 37.0, tvq = 9.0,

exp_crcl = 1.0, ΩCL = 0.3, σ_prop = 0.4)

nlme_fit = fit(nlme_model, data4nlme, init_param, Pumas.LaplaceI(),

constantcoef=(tvq=9.064, tvvp=37.5, exp_crcl=1.0,))
